# Supplementary material for: Fecal Microbiota Transplantation in the Treatment of Chronic Pouchitis: A Systematic Review
Source: Microorganisms. 2020 Sep 18;8(9):1433. doi: 10.3390/microorganisms8091433 (PMC7565999; doi:10.3390/microorganisms8091433)
Supplement: Supplementary file 1 [file microorganisms-08-01433-s001.pdf]

SUPPLEMENTARY FILE

Supplementary table 1. Search strategy

Search History 15th of April 2020

Medline (PubMed)
(((("Fecal Microbiota Transplantation"[Mesh]) OR fmt[Text Word]) OR (((fecal OR faecal OR bacteria* OR feces OR faeces OR stool OR intestinal OR microbiota OR microflora)) AND (transfer* OR transplant OR transplantations OR transplantation OR infusion*)))) AND (((("Pouchitis"[Mesh]) OR "Colonic Pouches"[Mesh]) OR "Proctocolectomy, Restorative"[Mesh]) OR ((pouch* OR IPAA OR "j pouch")))

The search resulted in 465 hits.

EMBASE

No.	Query	Results
#10	#4 AND #9	394
#9	#5 OR #6 OR #7 OR #8	35,267
#8	pouch* OR ipaa OR 'j pouch'	31,904
#7	'proctocolectomy'/exp	6,104
#6	'ileum pouch'/exp	1,578
#5	'ileoanal anastomosis'/exp	1,966
#4	#1 OR #2 OR #3	146,328
#3	(fecal OR faecal OR bacteria* OR feces OR faeces OR stool OR intestinal OR microbiota OR microflora) AND (transfer* OR transplant OR transplantations OR transplantation OR infusion*)	144,311
#2	fmt	3,928
#1	'fecal microbiota transplantation'/exp	3,683

Cochrane Central Register of Controlled Trials Library

ID	Search	Hits
#1	MeSH descriptor: [Fecal Microbiota Transplantation] explode all trees	37
#2	fmt	429
#3	(fecal OR faecal OR bacteria* OR feces OR faeces OR stool OR intestinal OR microbiota OR microflora) AND (transfer* OR transplant OR transplantations OR transplantation OR infusion*)	6,412
#4	#1 OR #2 OR #3	6,475
#5	MeSH descriptor: [Pouchitis] explode all trees	39
#6	MeSH descriptor: [Colonic Pouches] explode all trees	51
#7	MeSH descriptor: [Proctocolectomy, Restorative] explode all trees	110
#8	pouch* OR IPAA OR "j pouch"	1,033
#9	#5 OR #6 OR #7 OR #8	1,053
#10	#4 AND #9	33

The search resulted in 11 Cochrane Reviews, 1 Cochrane Protocol, and 21 Trials

Further search:
21st of April 2020 Web of Science (https://clarivate.com/webofsciencegroup/solutions/web-of-science/), U.S. National Library of Medicine. Clinicaltrials.Gov (https://clinicaltrials.gov/), World Health Organisation. International clinical trials registry platform (ICTRP) (https://apps.who.int/trialsearch/) and Opengrey System for Information in Grey Literature in Europe (http://opengrey.eu/) were assessed to look for unpublished data and further studies. This did not result in any other results or studies to include in the systematic review.

Supplementary table 2. Quality assessment of controlled, interventional studies

Cochrane collaboration's tool for assessing the risk of bias: Randomised controlled trials of faecal microbiota transplantation given by capsules:

	**Herfarth, 2019**
**Random sequence generation**	+
**Allocation concealment**	?
**Blinding of participants and personnel**	?
**Blinding of outcome assessment**	?
**Incomplete outcome data**	+
**Selective reporting**	?
**Other bias**	?

Legend:
+ = low risk of bias
? = unclear risk of bias
- = high risk of bias

Supplementary table 3. Quality assessment of cohort studies (full text and abstracts)

Author and year	1 (Objective)	2 (Population)	3 (Participation rate)	4 (eligibility criteria)	5 (sample size justification)	6 (exposure assessment)	7 (timeframe)	8 (exposure level)	9 (independent variables)	10 (assessed more than once)	11 (outcome)	12 (assessors blinded)	13 (loss to follow-up)	14 (statistically adjusted)	Quality rating (good, fair, poor)
Landy, 2015	1	1	1	1	0	1	1	0	1	0	1	0	1	0	fair
Selvig, 2020	0	1	1	0	0	1	1	0	1	0	1	1	1	0	fair
Kousgaard, 2020	1	1	1	1	1	1	1	0	1	1	1	0	0	0	good

***According to NHLBI quality assessment tool for cohort studies:**
1. Was the research question or objective in this paper clearly stated?
2. Was the study population clearly specified and defined?
3. Was the participation rate of eligible persons at least 50%?
4. Were all the subjects selected or recruited from the same or similar populations (including the same time period)? Were inclusion and exclusion criteria for being in the study prespecified and applied uniformly to all participants?
5. Was a sample size justification, power description, or variance and effect estimates provided?
6. For the analyses in this paper, were the exposure(s) of interest measured prior to the outcome(s) being measured?
7. Was the timeframe sufficient so that one could reasonably expect to see an association between exposure and outcome if it existed?
8. For exposures that can vary in amount or level, did the study examine different levels of the exposure as related to the outcome (e.g., categories of exposure, or exposure measured as continuous variable)?
9. Were the exposure measures (independent variables) clearly defined, valid, reliable, and implemented consistently across all study participants?
10. Was the exposure(s) assessed more than once over time?
11. Were the outcome measures (dependent variables) clearly defined, valid, reliable, and implemented consistently across all study participants?
12. Were the outcome assessors blinded to the exposure status of participants?
13. Was loss to follow-up after baseline 20% or less?
14. Were key potential confounding variables measured and adjusted statistically for their impact on the relationship between exposure(s) and outcome(s)?

**** Good if 10-14 points, fair if 5-9 points, poor if 0-4 points**

Note: 1 = Yes, 0 = No, CD = cannot determine, NA = not applicable, NR = not reported

Supplementary table 4. Quality assessment of case-series (full text and abstracts)

Author and year	1 (Objective)	2 (Population)	3 (consecutive)	4 (comparable)	5 (intervention)	6 (outcomes)	7 (length of follow up – 4 weeks [28 days])	8 (statistical methods)	9 (results)	Quality rating (good, fair, poor)
Fang, 2016	0	1	0	0	1	1	1	0	1	fair
Nishida, 2019	1	1	1	0	1	1	1	0	1	good
Schmid, 2017	0	0	0	0	1	0	1	0	0	poor
Stallmach, 2016	0	1	1	1	0	0	1	0	1	fair
Steube, 2017	0	0	1	0	0	1	1	0	0	poor

***According to NHLBI quality assessment tool for case series:**
1. Was the study question or objective clearly stated?
2. Was the study population clearly and fully described, including a case definition?
3. Were the cases consecutive?
4. Were the subjects comparable?
5. Was the intervention clearly described?
6. Were the outcome measures clearly defined, valid, reliable, and implemented consistently across all study participants?
7. Was the length of follow-up adequate?
8. Were the statistical methods well-described?
9. Were the results well-described?

**** Good if 7-9 points, fair if 4-6 points, poor if 0-3 points**

Note: 1 = Yes, 0 = No, CD = cannot determine, NR = not reported

PRISMA CHECKLIST

	#	Checklist item	Reported on page #
**TITLE**			
Title	1	Identify the report as a systematic review, meta-analysis, or both.	Page 1
**ABSTRACT**			
Structured summary	2	Provide a structured summary including, as applicable: background; objectives; data sources; study eligibility criteria, participants, and interventions; study appraisal and synthesis methods; results; limitations; conclusions and implications of key findings; systematic review registration number.	Page 2
**INTRODUCTION**			
Rationale	3	Describe the rationale for the review in the context of what is already known.	Page 4-5
Objectives	4	Provide an explicit statement of questions being addressed with reference to participants, interventions, comparisons, outcomes, and study design (PICOS).	Page 5
**METHODS**			
Protocol and registration	5	Indicate if a review protocol exists, if and where it can be accessed (e.g., Web address), and, if available, provide registration information including registration number.	Page 6
Eligibility criteria	6	Specify study characteristics (e.g., PICOS, length of follow-up) and report characteristics (e.g., years considered, language, publication status) used as criteria for eligibility, giving rationale.	Page 6
Information sources	7	Describe all information sources (e.g., databases with dates of coverage, contact with study authors to identify additional studies) in the search and date last searched.	Page 6
Search	8	Present full electronic search strategy for at least one database, including any limits used, such that it could be repeated.	Supplementary File, table 1
Study selection	9	State the process for selecting studies (i.e., screening, eligibility, included in systematic review, and, if applicable, included in the meta-analysis).	Page 6
Data collection process	10	Describe method of data extraction from reports (e.g., piloted forms, independently, in duplicate) and any processes for obtaining and confirming data from investigators.	Page 6
Data items	11	List and define all variables for which data were sought (e.g., PICOS, funding sources) and any assumptions and simplifications made.	Page 6-7

Risk of bias in individual studies	12	Describe methods used for assessing risk of bias of individual studies (including specification of whether this was done at the study or outcome level), and how this information is to be used in any data synthesis.	Page 8
Summary measures	13	State the principal summary measures (e.g., risk ratio, difference in means).	Page 8
Synthesis of results	14	Describe the methods of handling data and combining results of studies, if done, including measures of consistency (e.g., I^2) for each meta-analysis.	Page 8

Section/topic	#	Checklist item	Reported on page #
Risk of bias across studies	15	Specify any assessment of risk of bias that may affect the cumulative evidence (e.g., publication bias, selective reporting within studies).	Not applicable
Additional analyses	16	Describe methods of additional analyses (e.g., sensitivity or subgroup analyses, meta-regression), if done, indicating which were pre-specified.	Not applicable
**RESULTS**			
Study selection	17	Give numbers of studies screened, assessed for eligibility, and included in the review, with reasons for exclusions at each stage, ideally with a flow diagram.	Page 7 and figure 1
Study characteristics	18	For each study, present characteristics for which data were extracted (e.g., study size, PICOS, follow-up period) and provide the citations.	Table 2 and 3
Risk of bias within studies	19	Present data on risk of bias of each study and, if available, any outcome level assessment (see item 12).	Page 18 and Supplementary file table 3-4
Results of individual studies	20	For all outcomes considered (benefits or harms), present, for each study: (a) simple summary data for each intervention group (b) effect estimates and confidence intervals, ideally with a forest plot.	Table 2 and 3
Synthesis of results	21	Present results of each meta-analysis done, including confidence intervals and measures of consistency.	No meta-analysis performed
Risk of bias across studies	22	Present results of any assessment of risk of bias across studies (see Item 15).	Not applicable
Additional analysis	23	Give results of additional analyses, if done (e.g., sensitivity or subgroup analyses, meta-regression [see Item 16]).	Not applicable
**DISCUSSION**			
Summary of evidence	24	Summarize the main findings including the strength of evidence for each main outcome; consider their relevance to key groups (e.g., healthcare providers, users, and policy makers).	Page 19

Limitations	25	Discuss limitations at study and outcome level (e.g., risk of bias), and at review-level (e.g., incomplete retrieval of identified research, reporting bias).	Page 19-20
Conclusions	26	Provide a general interpretation of the results in the context of other evidence, and implications for future research.	Page 25
**FUNDING**			
Funding	27	Describe sources of funding for the systematic review and other support (e.g., supply of data); role of funders for the systematic review.	Page 32

From: Moher D, Liberati A, Tetzlaff J, Altman DG, The PRISMA Group (2009). Preferred Reporting Items for Systematic Reviews and Meta-Analyses: The PRISMA Statement. PLoS Med 6(7): e1000097. doi:10.1371/journal.pmed1000097

For more information, visit: **www.prisma-statement.org**.
